# 

**Published:** 1859-04

**Authors:** 


#### SUBSCRIPTIONS RECEIVED.

Drs. A. B. Wallace, Ga., $3 ; B. F. Bomar, Ga., $8 ; J. J. Robertson, S. C., $2 ; R. Searcy, Ga., $6 ; Hunter & Gamble, Ga., $3 ; O. W. Shepard, Ala., $3 ; Jubal Carpenter, Ala., $2 ; W. Burch, Ga., $3 ; Wm. H. Blalock, Ga., $10 ; J. P. Fitzgerald, Ala., $3 ; —— Watkins, Ga., $3 ; W. T. Burgamy, Ala., $3.

